# Yellow wave: impact of a structured educational initiative on trauma education development

**DOI:** 10.1590/acb411126

**Published:** 2026-03-09

**Authors:** Vitor Favali Kruger, João Victor Franqueira, Andrea de Melo Alexandre Fraga, Thiago Rodrigues Araujo Calderan, José Vitor Coimbra Trindade, Gustavo Pereira Fraga

**Affiliations:** 1 Universidade Estadual de Campinas – Faculdade de Ciências Médicas – Departamento de Cirurgia – Campinas (SP), Brazil.; 2 Universidade Estadual de Campinas – Faculdade de Ciências Médicas – Campinas (SP), Brazil.; 3 Universidade Estadual de Campinas – Faculdade de Ciências Médicas – Departamento de Pediatria – Campinas (SP), Brazil.

**Keywords:** Education, Medical, Mentoring, Students, Medical, Educational Measurement, General Surgery

## Abstract

**Purpose:**

To evaluate the impact of the Yellow Wave Initiative on trauma surgery education, academic skills, career choices, and educational equity among medical students from three Brazilian medical schools.

**Methods:**

This prospective longitudinal study enrolled 58 medical students from three institutions in Campinas, SP, Brazil. The intervention consisted of scientific mentorship, research methodology training, manuscript development for the 23rd European Congress of Trauma, and presentation skills workshops. Academic development, research capabilities, career intentions, and program satisfaction were assessed through post-congress electronic questionnaires using McNemar tests for paired comparisons and χ^2^ tests for associations.

**Results:**

Trauma league members showed higher interest in general surgery compared to non-members (61.5 *vs.* 22.7%, *odds ratio* = 10.88, *p* = 0.002). Significant improvements were observed across key academic skills post-intervention (all *p* < 0.001), including presentation abilities (from 20.8% good/very good pre to 85.4% post), research capabilities (25 to 79.2%), and academic writing (20.8 to 83.3%). Additionally, 62% of participants reported positive impact on their academic career aspirations (*p* = 0.006).

**Conclusion:**

The initiative enhanced academic skills and career development among Brazilian medical students, increasing engagement in trauma surgery and prevention activities while addressing educational gaps through hands-on learning.

## Introduction

Injuries account for 8% of global deaths, resulting in 4.4 million fatalities annually, primarily from road traffic collisions (33%), suicide (17%), and homicide (10%)^1^. Non-fatal injuries impose a substantial burden, particularly in low- and middle-income countries, necessitating improved trauma education and prevention strategies. To address gaps in trauma surgery education, the Yellow Wave Initiative (YWI) was developed as a collaborative program among three medical schools in Campinas, SP, Brazil. This initiative involved medical students and residents in producing scientific papers for an international congress through structured mentorship and research activities.

The purpose of this study was to evaluate the impact of YWI on participants’ academic writing skills, research capabilities, presentation abilities, career choices, awareness of preventive programs, and understanding of educational equity. We hypothesized that this structured educational intervention would significantly enhance professional development and influence career trajectories in trauma surgery.

## Methods

This prospective longitudinal observational study was approved by the Ethics in Research Committee of the Universidade Estadual de Campinas (UNICAMP)—Certificado de Apresentação para Apreciação Ética: 84544624.1.0000.5404; approval date: December 3, 2023. Written informed consent was obtained from all participants prior to enrollment, with detailed explanations of the study’s objectives, procedures, potential risks, and benefits provided via e-mail. Participation was voluntary, and participants could withdraw at any time without consequences. All data were anonymized, stored securely on password-protected servers, and accessed only by the research team, in compliance with the Declaration of Helsinki and Brazilian ethical guidelines.

The study assessed the impact of a structured academic program on trauma surgery education and research among 58 participants from three medical schools in Campinas: UNICAMP, Pontifícia Universidade Católica de Campinas (PUCC), and Faculdade São Leopoldo Mandic (SLM). These institutions were selected for their collaborative learning environments and established trauma leagues. The study population comprised medical students (third to sixth year) and general surgery residents whose abstracts were accepted for the 23rd European Congress of Trauma in Estoril, Portugal (October 2023). Inclusion criteria included enrollment in one of the participating schools, submission and acceptance of a congress abstract related to trauma or medical education, and availability to complete the program. Exclusion criteria were non-acceptance of abstracts or inability to participate due to academic conflicts. Participants were recruited voluntarily through institutional e-mails and trauma league announcements, with no incentives provided.

**Figure 1:**
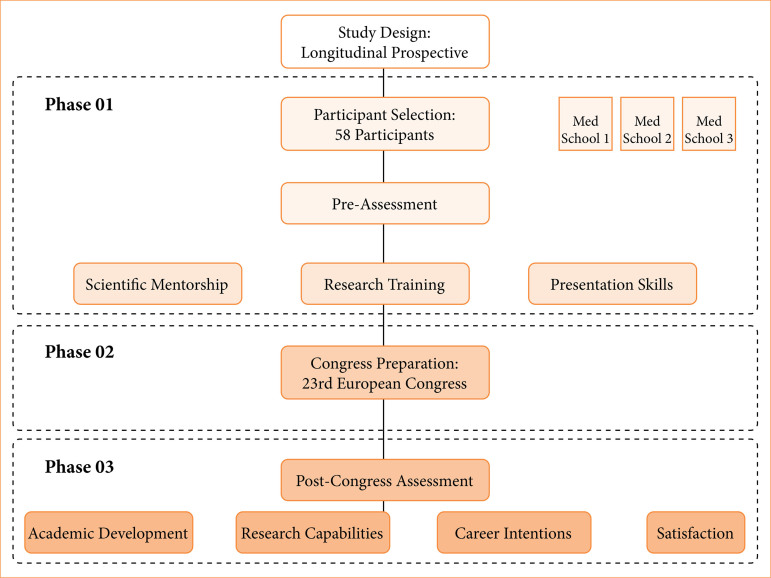
Overview of the Yellow Wave Initiative phases, including pre-congress preparation, congress activities, and post-congress evaluation.

Source: Elaborated by the authors.

The initiative encompassed three phases (Fig. 1). Phase 1 (pre-congress, May to September 2023) involved structured scientific mentorship, research methodology training (*e.g.*, conducting systematic literature reviews using Preferred Reporting Items for Systematic reviews and Meta-Analyses guidelines and collecting clinical data from institutional databases), and presentation skills development through workshops. Participants, under faculty supervision, prepared abstracts for international submission. Phase 2 (congress activities, October 2023) included oral or poster presentations, panel discussions, and networking opportunities. Phase 3 (post-congress assessments, November 2023) focused on evaluation. The program adopted a competency-based education framework, pairing experienced faculty mentors with students to develop key skills in a collaborative model.

Participant competencies were assessed using a retrospective pre-post design (also known as then-test methodology), wherein participants rated their academic skills both before and after the YWI within a single anonymous electronic questionnaire administered 30 days post-congress. This approach was selected to ensure uniform reference frames for self-assessment and practical feasibility in the educational context, as participants evaluated their before and after states using the same internal criteria developed through the learning experience. The questionnaire, pilot-tested with five non-participating students, assessed five domains: academic development, research capabilities, career intentions, social engagement, and program satisfaction. It included 5-point Likert scales for paired pre-post comparisons (*e.g.*, Rate your academic writing skills before AND after the initiative: 1 = poor to 5 = excellent) and standardized open-ended questions. The 30-day post-congress timeframe and anonymous data collection (via Google Forms; Suppl. Mat. 1) balanced memory retention with completion feasibility while minimizing recall and social desirability bias.

Primary outcomes were changes in academic skills (writing, research, presentation), measured via Likert scale shifts. Secondary outcomes included social engagement (*e.g.*, participation in trauma prevention activities) and knowledge accessibility indicators (*e.g.*, socioeconomic equity in participation).

Statistical analyses were performed using SAS System for Windows (version 9.4). Descriptive statistics summarized data as frequencies, percentages, means ± standard deviations. Associations between categorical variables (*e.g.*, trauma league membership and career interest) were evaluated using χ^2^ tests, with *odds ratios* (OR) and 95% confidence intervals (95%CI) calculated. Paired before-after comparisons of dichotomized self-rated competencies (poor/fair *vs*. good/very good/excellent) employed McNemar tests. Proportional analyses assessed participation patterns across socioeconomic strata. Qualitative data were analyzed through content analysis by two independent researchers, with themes identified inductively and discrepancies resolved by consensus. Statistical significance was defined as *p* < 0.05. This study conforms to the STrengthening the Reporting of OBservational studies in Epidemiology (STROBE) statement^2^ (Suppl. Mat. 2).

## Results

The YWI enrolled 58 participants, including medical students from the third to sixth years and general surgery residents, with 48 completing the post-congress questionnaire (response rate = 82.8%). Participants had a mean age of 24.3 ± 2.1 years old, and 56.2% were female (n = 27). Institutional distribution was predominantly UNICAMP (72.9%, n = 35), followed by PUCC (22.9%, n = 11) and SLM (4.1%, n = 2). Among the 18 abstracts submitted, 16 were accepted (88.9%), comprising 15 posters and one oral presentation; 75% (n = 12) focused on medical education and 25% (n = 4) on trauma and emergency surgery. All analyses were based on the 48 respondents.

Trauma surgery classes were attended by 89.5% of participants (n = 43), with 54.1% (n = 26) involved in trauma leagues. Importantly, trauma league members demonstrated significantly higher interest in general surgery careers compared to non-members (61.5 vs. 22.7%; OR = 10.88, 95%CI 2.9–40.7, *p* = 0.002), indicating a nearly 11-fold increased likelihood. League participation also correlated significantly with greater research engagement (*p* = 0.028) and prevention activities (*p* = 0.038), as detailed in Table 1.

**Table 1:** Association between trauma league participation and career choice (n = 48).

Characteristics	Yes n (%)	No n (%)	*p* -value^a^	OR (95%CI)
**League status**				
League members	16 (61.5)	5 (19.2)	0.002	10.88 (2.9–40.7)
Non-members	5 (22.7)	17 (77.3)		
**Associated outcomes**				
Research engagement	22 (84.6)	12 (54.5)	0.028	–
Prevention activities	25 (96.2)	17 (77.3)	0.038	–

a χ^2^ = 9.43; OR: *odds ratio*; 95%CI: 95% confidence interval.

Source: Elaborated by the authors.

Most participants were fourth- or fifth-year students (54.1%, n = 26), followed by third-year students (20.8%, n = 10), residents (12.5%, n = 6), sixth-year students (8.3%, n = 4), and multidisciplinary staff (4.1%, n = 2). Socioeconomic analysis revealed a predominance of higher- and middle-income participants (81.2%), with 79.1% relying on family financial support (*p* < 0.001; Table 2).

**Table 2:** Income distribution and financial support of participants (n = 48).

Variables	n (%)	*p* -value
**Income distribution**		**< 0.001^a^**
Higher income (> $ 4,631.00)	20 (41.67)	
Middle-income ($ 1,494.00–$ 4,631.00)	19 (39.58)	
Lower middle-income ($ 610.00–$ 1,494.00)	8 (16.67)	
Lower income (< $ 610.00)	1 (2.08)	
**Source of financial support**		**< 0.001^b^**
Parental or family support	38 (79.17)	
Own resources	4 (8.33)	
Scholarships	3 (6.25)	
Student loans	3 (6.25)	

a χ^2^ test for income distribution;

b χ^2^ for financial support sources.

Source: Elaborated by the authors.

**Figure 2:**
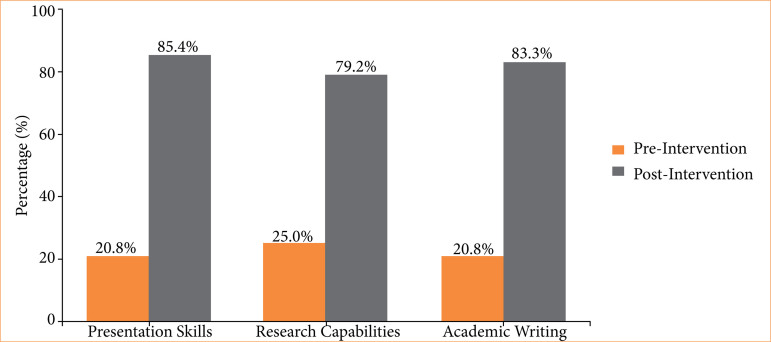
Pre/post-intervention skills improvement.

Source: Elaborated by the authors.

Significant improvements were observed across key academic skills post-intervention (all *p* < 0.001), including presentation abilities (from 20.8% good/very good pre to 85.4% post), research capabilities (25 to 79.2%), and academic writing (20.8 to 83.3%), underscoring the program’s efficacy in scholarly development (Fig. 2). Self-rated competencies are showed in Table 3.

**Table 3:** Self-rated competencies before and after the yellow wave initiative (n = 48)*.

Competency	Pre-initiative good/very good	Post-initiative good/very good	Change	McNemar
	n (%)	n (%)	(pp)	*p* -value
Presentation skills	10 (20.8)	41 (85.4)	+64.6	< 0.001
Research capabilities	12 (25.0)	38 (79.2)	+54.2	< 0.001
Academic writing	10 (20.8)	40 (83.3)	+62.5	< 0.001

* Competencies rated on a 4-point scale (fair, regular, good, very good). Values represent participants rating themselves as “good” or “very good” (versus “fair” or “regular”). McNemar test used for paired comparisons; pp = percentage points.

Source: Elaborated by the authors.

Interprofessional components yielded strong outcomes, with 95% of participants engaging in trauma prevention activities and 52.5% in more than five such events. The Prevent Alcohol and Risk-related Trauma in Youth (P.A.R.T.Y.) program was most common (79%), followed by mass casualty simulations (45%), and trauma symposia (30%). Reported challenges during abstract preparation included situational anxiety (35%), language barriers (24%), topic unfamiliarity (18%), and limited presentation experience (16%), while 7% faced none.

Career aspirations showed notable shifts, with 62% reporting positive influence on academic trajectories (*p* = 0.006) and high intentions for continued research (81.3%, *p* < 0.001) and international submissions (83.3%, *p* < 0.001), among other impacts (Table 4).

**Table 4:** Impact of educational experience on academic development and career intentions (n = 48).

Variables	Yes n (%)	No n (%)	p-value^a^
**Professional development**			
Changed view on trauma prevention	45 (93.8)	3 (6.2)	**< 0.001**
Recommend trauma league	44 (91.7)	4 (8.3)	**< 0.001**
Enhanced teamwork/leadership	39 (81.3)	9 (18.7)	**< 0.001**
Continued medical research	40 (83.3)	8 (16.7)	**< 0.001**
**Career and research intentions**			
Interest in international fellowship	35 (72.9)	13 (27.1)	**0.001**
Submit international papers	34 (70.8)	14 (29.2)	**0.002**
Academic career influence	33 (68.8)	15 (31.2)	**0.006**
International networking	33 (68.8)	15 (31.2)	**0.006**
Residency in general surgery	31 (64.6)	17 (35.4)	**0.005**
Research in trauma surgery	25 (52.1)	23 (47.9)	0.371

a χ^2^ test.

Source: Elaborated by the authors.

## Discussion

The YWI introduces a collaborative, mentorship-driven model for trauma surgery education in Brazil, integrating interprofessional research, experiential learning, and financial support to promote educational equity and address curriculum gaps. Unlike traditional didactic programs, YWI engaged students from three Campinas medical schools in producing and presenting scientific work at an international congress, fostering skill development and surgical interest while emphasizing prevention and global networking.

This experiential approach aligns with frameworks shown superior for clinical competencies in studies on extracurricular leagues^3,4^. Brazil hosts 437 medical schools as of 2025—the second highest globally after India’s 605—, despite India’s population being approximately 6.6 times larger, with over 79% private institutions raising quality concerns in emergency trauma training^5^. YWI’s integration of adapted programs like P.A.R.T.Y. from Toronto, Canada, implemented locally since 2010 with broad community outreach, exemplifies how student-led efforts surpass classroom methods in engagement and awareness, consistent with evaluations of similar initiatives^6,7^. Participation skewed toward mid-curriculum years mirrors literature on reduced extracurricular activity in later stages due to exam focus. YWI’s novelty includes promoting multidisciplinary involvement, countering barriers like workloads that restrict non-physicians, as systematic reviews demonstrate improved outcomes through such engagement^8^.

This underscores institutional needs for inclusive research teams, extending beyond benefits in emergency nursing. Socioeconomic disparities reflect Brazil’s inequities, and economic factors hinder access; YWI’s aid for lower-income participants enabled merit-based achievements, supporting theories that equitable knowledge reduces inequalities^9^.

The YWI’s mentorship accelerated development beyond conventional models, influencing aspirations amid global surgical recruitment declines. As a Brazilian innovation, trauma leagues—linked to superior cognitive and clinical performance—offer solutions through competency-based training^10^. Despite opportunities, general surgery faces challenges, with historically unfilled residency positions in the United States of America and patterns in diverse countries over decades^11–15^. Landmark surveys highlight low trauma interest due to non-operative demands and patient complexities, yet integrated models like Louisville’s emphasize early exposure for commitment^16,17^. YWI echoes that leagues shaped specialty choices, attributing surgical decisions to experiential involvement. Unfortunately, in Brazil, trauma surgery is not officially recognized as a specialty, representing a significant barrier to both trauma-care quality and medical education. This gap is particularly concerning as approximately 70% of healthcare professionals working in emergency departments manage trauma patients during their careers^18^. Hindawi et al.^19^ reported that lower- and middle-income countries face persistent challenges in surgical care delivery, including inadequate infrastructure, limited availability of trained surgical personnel, lack of essential medical equipment, and insufficient financial resources allocated to healthcare. Brazil’s failure to recognize trauma surgery as a specialty illustrates how these systemic barriers manifest in practice, directly impacting the quality and development of emergency surgical services.

This study employed a retrospective pre-post assessment design, which may introduce response-shift bias and recall limitations. However, this approach offers advantages for educational evaluation: the response-shift effect itself reflects metacognitive development, and retrospective designs ensure uniform reference frames as participants use consistent internal standards to evaluate their competencies. Additionally, post-congress questionnaires were administered after participants had already produced their abstracts, potentially influencing their self-assessment. Strategic design elements—anonymous data collection, 30-day post-congress timing, and pilot-tested prompts—were implemented to minimize social desirability bias. The substantial effect sizes across all domains and high completion rate (86.8%) support the validity of reported gains. Retrospective pre-post designs are established in health professions education literature when prospective baseline assessment is impractical. Future studies should incorporate objective measures such as publication rates or independent competency assessments to complement self-reported data.

## Conclusion

The YWI represents a competency-based educational model that positively impacts Brazilian medical students’ academic development and career choices. Through structured mentorship and hands-on activities, the program enhances scholarly skills, boosts interest in surgical specialties, and promotes engagement in trauma prevention, thereby addressing gaps in trauma education and offering a replicable framework for innovation in resource-constrained environments.

## Data Availability

Supplementary Material 1: https://docs.google.com/forms/d/e/1FAIpQLSfKg1G4NNkalT1WYQ93nXDDkaha8BQlzr3cPTuhY648_vf6qA/closedform. Supplementary Material 2: https://doi/org/10.6084/m9.figshare.31272658.

## References

[1] World Health Organization (2022). Preventing injuries and violence: an overview.

[2] von Elm E, Altman DG, Egger M, Pocock SJ, Gøtzsche PC, Vandenbroucke JP, STROBE Initiative (2007). The Strengthening the Reporting of Observational Studies in Epidemiology (STROBE) statement: guidelines for reporting observational studies. Lancet.

[3] Simões RL, Bermudes FA, Andrade HS, Barcelos FM, Rossoni BP, Miguel GP, Fraga GP (2014). Trauma leagues: an alternative way to teach trauma surgery to medical students. Rev Col Bras Cir.

[4] Simões RL, Bicudo AM, Passeri SMRR, Calderan TRA, Rizoli S, Fraga GP (2023). Can trauma leagues contribute to better cognitive performance and technical skills of medical students?. Eur J Trauma Emerg Surg.

[5] Scheffer M (2025). Demografia médica no Brasil 2025.

[6] Banfield J, Gomez M, Kiss A, Redelmeier DA, Brenneman F (2011). Effectiveness of the P.A.R.T.Y. (Prevent Alcohol and Risk-related Trauma in Youth) program in preventing traumatic injuries: a 10-year analysis. J Trauma.

[7] Dorigatti AE, Jimenez LS, Redondano BR, Carvalho RB, Calderan TR, Fraga GP (2014). Importance of multidisciplinary trauma prevention program for youth. Rev Col Bras Cir.

[8] Zavotsky KE, Wolf LA, Baker KM, Carman MJ, Clark PR, Langkeit K, Dunn SL, Gettner P, Gregory S, Handley CM, Harmon KJ, Hernandez CA, Moser DK, Munro CL, Padden ML, Schiech L, Shiao SYK (2014). Benefits to ED nurses participating in interdisciplinary research. J Emerg Nurs.

[9] Tilly C. (2007). Unequal access to scientific knowledge. J Hum Dev Capab.

[10] Simões RL, Dorigatti AE, Silveira HJV, Calderan TRA, Rizoli S, Fraga GP (2018). Trauma leagues-a novel option to attract medical students to a surgical career. World J Surg.

[11] Kelly E, Rogers SO Jr (2012). Graduate medical education in trauma/critical care and acute care surgery: defining goals for a new workforce. Surg Clin North Am.

[12] Fischer JE (2007). The impending disappearance of the general surgeon. JAMA.

[13] Ito Y (2008). Surgical education and postgraduate training in Japan. World J Surg.

[14] Makama JG, Ameh EA (2010). Does general surgery clerkship make a future career in surgery more appealing to medical students?. Afr Health Sci.

[15] Kaderli R, Buser C, Stefenelli U, Businger A (2011). Students’ interest in becoming a general surgeon before and after a surgical clerkship in German-speaking Switzerland. Swiss Med Wkly.

[16] Richardson JD, Miller FB (1992). Will future surgeons be interested in trauma care? Results of a resident survey. J Trauma.

[17] Richardson JD, Miller FB, Polk HC (1993). Can we improve the surgical manpower for providing trauma care? A model for success. J Trauma.

[18] Chalmers I, Hedges LV, Cooper H (2002). A brief history of research synthesis. Eval Health Prof.

[19] Hindawi MD, Isik A, Rosa F, Visconti D, Nechay T, Chowdhury S, Demetrashvili Z, Tan E, Sganga G, Sartelli M, Podda M, Di Saverio S, Bala M, Fugazzola P, Rausei S, Kluger Y, Ten Broek R, Biffl WL, Leppaniemi A, Kirkpatrick AW, Kaafarani HMA, Tolonen M, Weber DG, Kafka-Ritsch R, Tartaglia D, Amico F, Beka SG, Ioannidis O, Bouliaris K, Karamarkovic A, Augustin G, Kelly MD, de’Angelis N, Fette A, Hecker A, Reichert M, Chirica M, Bensard DD, Picetti E, Ivatury RR, Litvin A, Shelat VG, Negoi I, Cui Y, Coimbra R, Moore EE, Catena F (2025). Global perspectives in acute and emergency general surgery in low and middle-income countries: a WSES project protocol for scoping review on global surgery. World J Emerg Surg.

